# The first arriving virus shapes within-host viral diversity during natural epidemics

**DOI:** 10.1098/rspb.2023.1486

**Published:** 2023-09-13

**Authors:** Maija Jokinen, Suvi Sallinen, Mirkka M. Jones, Jukka Sirén, Emy Guilbault, Hanna Susi, Anna-Liisa Laine

**Affiliations:** ^1^ Department of Evolutionary Biology and Environmental Studies, University of Zürich, 8057 Zürich, Switzerland; ^2^ Organismal and Evolutionary Biology Research Programme, Faculty of Biological and Environmental Sciences, University of Helsinki, PO Box 65, 00014, Finland; ^3^ Institute of Biotechnology, HiLIFE-Helsinki Institute of Life Science, University of Helsinki, PO Box 65, 00014, Finland

**Keywords:** virus ecology, priority effects, sequential infection, co-infection

## Abstract

Viral diversity has been discovered across scales from host individuals to populations. However, the drivers of viral community assembly are still largely unknown. Within-host viral communities are formed through co-infections, where the interval between the arrival times of viruses may vary. Priority effects describe the timing and order in which species arrive in an environment, and how early colonizers impact subsequent community assembly. To study the effect of the first-arriving virus on subsequent infection patterns of five focal viruses, we set up a field experiment using naïve *Plantago lanceolata* plants as sentinels during a seasonal virus epidemic. Using joint species distribution modelling, we find both positive and negative effects of early season viral infection on late season viral colonization patterns. The direction of the effect depends on both the host genotype and which virus colonized the host early in the season. It is well established that co-occurring viruses may change the virulence and transmission of viral infections. However, our results show that priority effects may also play an important, previously unquantified role in viral community assembly. The assessment of these temporal dynamics within a community ecological framework will improve our ability to understand and predict viral diversity in natural systems.

## 1. Introduction

In recent years, largely due to advancements in metagenomic studies, there has been a rapid increase in the discovery of novel virus species. Metagenomic surveys have revealed high viral diversity in wild, uncultured habitats and the tremendous complexity of viral communities [1–4]. Despite this increasing knowledge of natural viral diversity, the drivers of viral community assembly are still poorly understood [5,6]. Insights into the mechanisms of viral community assembly are key to an improved understanding of virus ecology and disease dynamics.

Community assembly is expected to be influenced by abiotic filters, spatial structure and biotic interactions [7], and the limited evidence available to date indicates that this also holds true for viruses [8–11]. In many pathogens, changes in temperature and humidity impact their ability to infect, reproduce and transmit within and between their hosts [12–14]. Hence, it is perhaps unsurprising that the prevalence and diversity of several pathogen species—including viruses—follow elevational and latitudinal gradients [15–17]. The effects of climate drivers on viral transmission and activity have been notably studied with viruses that cause clinically significant respiratory tract infections [18,19] and in marine environments [20,21]. In addition to natural environmental variation, human actions modify the environment, directly and indirectly, through climate change [22], habitat fragmentation [23,24] and altered nutrient cycling [25,26], all of which can result in changes in host biodiversity [27]. Biotic factors potentially affecting viral community assembly comprise their hosts and vectors as well as other pathogens, including other virus species and different virus strains. Given that the host acts as the immediate environment for viruses, the spatial distribution of hosts and host diversity—in terms of both interspecific and intraspecific variation—are critical determinants of viral diversity [10,28,29]. For viruses transmitted by vectors, landscape structure and possible disturbance in the vector community may affect vector distribution and thus also viral transmission [30,31].

Virus–virus interactions, whether direct or mediated by the host, are potentially important determinants of viral community composition [32–34]. For example, viral infection in plants can increase vector attraction or alter vector feeding behaviour, indirectly enhancing viral transmission and viral co-occurrences [35–38]. Co-infections by multiple viruses or other pathogens in the same host are common and may result from simultaneous exposure, for example, to a common vector [39,40], intermediate host [41] or from the consumption of prey carrying multiple viruses [42]. However, co-infections are more often thought to establish as a result of sequential infections, where the intervals between infections and the arrival times of pathogens vary [43–45]. Priority effects describe the timing and order in which species arrive in an environment, and how the first arriving species at a site impacts subsequent community development [46,47]. The study of priority effects has its roots in community ecology, where it has been used to understand how species' order of arrival contributes to defining the patterns and relative abundances of co-occurring species [48,49]. As the host delineates a clear habitat into which viral communities assemble [50], priority effects provide an intuitive framework for host–virus and host–pathogen research [44,51–53].

Inhibitory priority effects describe situations in which an early-arriving species alters the environment such that the establishment of later-arriving species is either suspended or entirely inhibited [54]. Early colonists may, for example, induce the host's immune response, thus inhibiting or delaying infection by species arriving later [55,56]. Inhibitory effects may also be caused by disease symptoms, such as necrosis of host tissue, which render the host environment unfavourable for colonization by other species [46]. In agriculture, cross-protection arising from the deliberate infection of crops with a milder virus strain in order to induce immunity to subsequent infection by more severe strains, is viewed as a promising plant protection tool [55]. By contrast, facilitative priority effects are characterized by the early-arriving species modifying the host environment to be more favourable for later-arriving species. Defence against the first-arriving species can be costly for the host, leaving the host more susceptible to a secondary infection [57,58]. As an example of cross-kingdom pathogenic facilitative priority effects, respiratory tract infection and lung tissue damage caused by a virus often leads to secondary bacterial infection [59,60]. Furthermore, host immunity is probably an important mediator of priority effects, as recent research suggests that genotypes of the same species vary in their susceptibility to viruses and other pathogens that affect community structure [10,61,62]. By extension, different genotypes of the same host species may also vary in their resistance to sequential infections and in their tendency to harbour co-infections due to genetic differences in their immune responses [63,64].

Here, we investigate how viral infection at the beginning of seasonal epidemics affects subsequent viral community assembly. We used data on the early season viral infection status of host plants from a transplant experiment by Sallinen *et al*. [10] to select plant individuals that differ in their infection status thereby enabling us to assess how this affects subsequent virus community assembly. We focus on successive samples from these same host individuals collected later in the season that have not been previously analysed. Samples were analysed by PCR to detect five focal *Plantago lanceolata*-infecting viruses: *Plantago lanceolata latent virus* (PILV) [65]*, Plantago latent caulimovirus*, *Plantago lanceolata betapartitivirus*, *Plantago enamovirus* [66] and *Plantago closterovirus* [66]. The transplant experiment consisted of cloned naive replicates of four *Pl. lanceolata* genotypes, which were placed into wild *Pl. lanceolata* populations during seasonal viral epidemics. In their study, Sallinen *et al.* [10] found both host genotype and local population context to explain a large proportion of variation in early season virus occurrences.

To date, priority effects have not been studied experimentally during naturally occurring viral epidemics. In this study, we leverage the early season infection status data of Sallinen *et al.* [10] to explore evidence of priority effects of early arrivals on the late season occurrences of the five focal viruses. We fitted a joint species distribution model (JSDM) [67,68] to test for priority effects on viral community assembly, while controlling for host genotype and other host characteristics. Specifically, we ask: (i) Does the early infection status of the host shape subsequent viral occurrence patterns? (ii) Can we identify inhibitory or facilitative priority effects depending on which virus infected the host first? (iii) Are viral priority effects associated with particular host genotypes?

## 2. Material and methods

### (a) Study species

The host species, *Pl. lanceolata*, a perennial herbaceous plant, is an obligate out-crosser capable of reproducing both clonally by side rosettes and sexually [69]. *Plantago lanceolata* occurs worldwide. In the Åland Islands, southwest Finland, the plant forms a network consisting of approximately 4000 small fragmented populations [70]. These populations have been monitored since 1990 for the presence of *Melitaea cinxia* butterflies and the epidemiological dynamics of the *Pl. lanceolata* infecting fungus *Podosphaera plantaginis* have been studied since 2001 [70,71]. Host–virus interactions in the system have been studied since 2017, revealing diverse viral communities [10,33,65,66].

To investigate priority effects in viral community assembly during seasonal viral epidemics, we focused on five common, recently characterized viruses from the Åland Islands: DNA viruses *Plantago lanceolata latent virus* (PlLV) in the genus *Capulavirus* [65] and *Plantago latent caulimovirus* [66] in the genus *Caulimovirus*, and RNA viruses *Plantago betapartitivirus* in the genus *Betapartitivirus*, *Plantago enamovirus* in the genus *Enamovirus* and *Plantago closterovirus* in the genus *Closterovirus* [65,66]. For clarity, we will refer to these viruses hereafter as PlLV*, Caulimovirus*, *Closterovirus*, *Betapartivirus* and *Enamovirus.* The five focal viruses are among the most prevalent *Pl. lanceolata*-infecting viruses in the Åland system [33] and have been characterized by small RNA (sRNA) sequencing, which targets the small interfering RNA (siRNA), a plant immune response to viral infection [72]. Viruses from the genera *Capulavirus*, *Caulimovirus*, *Enamovirus* and *Closterovirus* are transmitted via aphid vectors [73–76] and are considered plant-specific, whereas viruses in the genus *Betapartitivirus* can infect both plants and fungi [77,78]. Symptoms associated with the five focal viruses are unclear, but can include yellowing, redness, curliness and necrotic lesions [66]. Susi *et al.* [66] linked PlLV infection with yellowing of the leaf.

### (b) Preparation of sentinel plant material and field experiment

To study priority effects during seasonal viral epidemics, we placed a total of 320 sentinel plants into four wild populations of *Pl. lanceolata* in the Åland Islands, Finland. To ensure genetically homogenous host material, 80 replicate sentinel plants were cloned from each of four greenhouse-grown maternal plants. The cloned individuals were assumed to represent four distinct *Pl. lanceolata* genotypes as the maternal plants originated from populations 7–40 km apart (genotype IDs: 609_19; 4_13; 511_14; 2929_6). In short, four-week-old maternal plants potted with a 1 : 1 proportion of potting soil and sand were placed on top of 11 cm × 11 cm pots filled with vermiculate. The plants and pots were then placed on trays filled with water. When roots had grown through to the bottom pot, the roots were cut and allowed to sprout within the bottom pot. Shoots from the cut roots were individually planted into new 10 cm × 10 cm pots and grown for two additional months in the greenhouse. For more details on cloning of the host material, see Sallinen *et al.* [10]. To exclude possible seed-derived viral infection, the maternal plants were confirmed to be free of the five target viruses by PCR.

In the last week of May 2017, the cloned individuals were placed into four wild *Pl. lanceolata* populations in the Åland Islands. The populations (ID:s 877, 9031, 433, 3302) are located in different parts of the Åland Islands and do not include the populations of origin of the maternal plants. We placed 80 cloned plants into each of the four populations, 20 replicates of each of the four maternal genotypes. The experiment hence included a total of 320 sentinel plant individuals. The plants were randomly placed within natural vegetation in the chosen meadows and separated from the local soil by placing the plant pots inside plastic boxes (approx. 13 cm × 11 cm). To minimize within-population spatial effects, the placement of the plants among the plastic boxes was shuffled three times per week. The plants were watered when needed.

Two weeks after introducing the sentinel plants into the wild populations, we recorded signs of herbivory (holes, bite marks and thrip damage), counted the number of leaves and measured the width and length of the largest leaf. Based on these measurements, we calculated plant size as *n × A*, where *n* is the number of leaves and *A =* π*ab*, where *a* is the half axis of the width of the largest leaf, and *b* is the half axis of the length of the largest leaf. For RNA and DNA extraction, 3 cm^2^ and 1 cm^2^ pieces of leaf tissue were collected, respectively. The samples for DNA extraction were kept in a cold bag and the sample for RNA extraction was snap frozen with liquid nitrogen on site. After the first sampling date, hereafter the early season timepoint, we repeated DNA and RNA sample collection at two-week intervals until the last week of July 2017 (sampling timepoints 2–4, hereafter T2-T4).

### (c) Nucleic acid extraction and virus detection in samples from the field experiment

To detect DNA or RNA of the target viruses from the leaf tissue samples, we first assigned the samples to nucleic acid extractions. Total DNA was extracted from a 1 cm^2^ leaf tissue sample with EZNA Plant Kit (Omega Biotek, USA) at the Institute of Biotechnology, University of Helsinki, Finland, following the manufacturer's instructions. The leaf tissue prior to extraction and the extracted total DNA were stored at −20°C. For total RNA extraction, we used a modified protocol from Chang *et al*. [79] with additional acid phenol (pH 4.5) clean-up steps [79]. In short, a 3 cm^2^ piece of leaf tissue was ground into a fine powder using liquid nitrogen, then 800 µl of warm extraction buffer was added, and the sample was mixed thoroughly. After this, 800 µl of acid phenol-chloroform-IAA solution (with ratio 25 : 24 : 1, respectively) was added and centrifuged at 13 500 rpm for 15 min in RT. The supernatant was transferred to a new tube and the clean-up step with acid phenol-chloroform-IAA solution was repeated. At the end of the extraction, the purified RNA pellet was resuspended in 25 µl of nuclease-free water. The leaf tissue prior to RNA extraction and the extracted RNA were stored at −80°C. The extracted RNA was translated into cDNA before PCR. For cDNA translation, 2 ng of RNA was combined with 2 µl of 50 µM random hexamer primers (Promega Corporation, USA), nuclease-free water was added to a final volume of 17.125 µl and the reaction was incubated at 70°C for 5 min. For the reverse transcription reaction, 1 µl of Moloney Murine Leukemia Virus Reverse Transcriptase (M-MLV RT; Promega Corporation, USA), 5 µl of M-MLV RT 5x buffer (Promega Corporation, USA), 1.25 of 10 mM dNTP mix (Thermo Fischer) and 0.625 µl of RiboLock RNase inhibitor (Thermo Fischer Scientific, USA) were added to the reaction mix and incubated for 60 min at 37°C.

To detect PlLV and *Caulimovirus* DNA and *Betapartitivirus, Enamovirus* and *Closterovirus* RNA from the samples, we used previously described primers for each virus [9,62,63]. Primer sequences can be found in the electronic supplementary material (table S1). The PCR reaction consisted of 500 nmol of the corresponding reverse and forward primers, 1 µl of template DNA or cDNA, 5 µl of GoTaq Green 5x Mastermix (Promega Corporation, USA) and nuclease-free water to a final reaction volume of 10 µl. The PCR conditions were initial denaturation at 95°C for 2 min, followed by 35 cycles at 95°C for 2 min, 50–60°C for 40 s and 72°C for 1 min. The final extension was done at 72°C for 5 min. A positive control and a water control were included in each PCR run. The sizes of the amplicons were analysed on 1.5% agarose gel, stained with GelRed (Biotium, USA) and visualized using the Bio-Rad Gel Doc XR + imaging system (Bio-Rad Laboratories, USA).

### (d) Statistical analysis

To investigate how host early season characteristics affect subsequent viral occurrence patterns, we pooled the detected occurrences of all five viruses from the three late season sampling dates (T2–T4) into a single late season timeperiod for the statistical analysis. Data pooling was done to obtain an observed viral community for each host individual from the late season and to maximize sample size, and hence statistical power over the late season timeperiod. To explore the effects of early season viral colonization and host genotype on late season viral occurrences in hosts, we ran a JSDM using the hierarchical modelling of species communities (HMSC) [67,80] framework. HMSC is a Bayesian hierarchical generalized linear mixed model, in which the responses of species to ecological variables are modelled with a combination of community- and species-level parameters [67]. In addition to the pooled model, we fitted models with the viral response data from each of the late season sampling timepoints (T2–T4) separately, to test whether the pooled model results were representative of these, or whether, for example, there is evidence that priority effects set in motion in the early summer either weaken or strengthen over time.

The response variables in our HMSC model were vectors of the late season occurrences of each of the five focal viruses recorded in the host individuals. As fixed effect predictors, we included (i) binary data on the early season occurrences of each of the three focal viruses detected at that timepoint (i.e. *Closterovirus*, *Betapartitivirus* and PlLV; see Results for details), (ii) host genotype, (iii) log-transformed host plant size, (iv) the presence/absence of herbivory and (v) the number of failed RNA samples, if any. The latter variable was included to account for minor differences among host plants in the likelihood of recording the late season presence of RNA viruses, arising from the fact that data on RNA virus presence (*Closterovirus*, *Betapartitivirus* and *Enamovirus*) was unavailable for 29 late season samples. As random effect predictors, we included (i) transplant population identity and (ii) individual host identity. Host individual identity was included as a random effect to allow us to quantify any residual (unexplained) structure in late season viral occurrences at the plant level. To account for any spatial dependence of observations in each of the four populations, we included population identity as a random effect. We sampled the posterior distribution with four independent Markov chain Monte Carlo (MCMC) chains, each run for 1 875 000 iterations, and discarded the first 625 000 as burn-in. The remaining iterations were thinned by 5000 to yield 250 posterior samples per chain, and thus 1000 posterior samples in total.

We examined model fit by evaluating both explanatory performance and predictive performance, assessed via 10-fold cross-validation, as a function of Tjur *R*^2^ (Tjur's coefficient of determination) [81] and AUC (area under the curve) [82]. We ran our analyses using the R-package Hmsc 3.0–13. [68]. For all analyses, we used R version 4.3.0. The statistical analysis pipeline and input data are available on GitHub (https://github.com/mirkkajones/ViralCommHmsc).

We addressed our first research question by exploring how much variation (%) in the late season occurrences of each five focal viruses in the host was explained by early season viral infection status using an extended variance partitioning summary from HMSC (mean and 95% credible interval). We addressed our second research question by exploring the strength and direction of the predicted effects of each early season viral covariate on late season viral occurrences, based on the beta parameters of the regression models. Positive and negative responses to the early season viral covariates with strong (greater than 95%) posterior support were considered as evidence of positive (facilitative) and negative (inhibitory) priority effects, respectively. We addressed the third question by exploring whether the predicted late season colonization patterns of each virus as a function of early season viral infections differed among the four host plant genotypes.

## 3. Results

### (a) Virus detection from the host individuals by PCR

**Figure 1.**
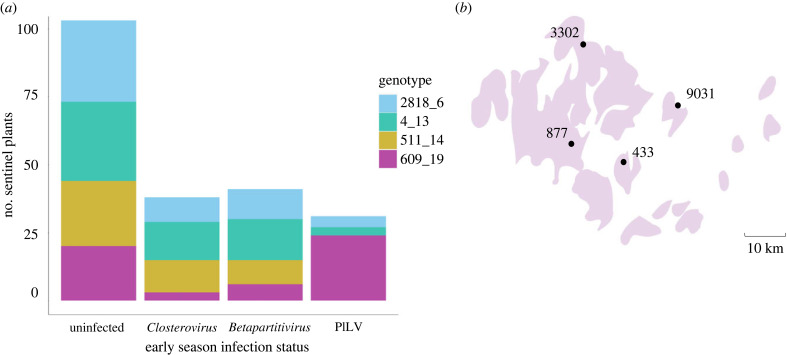
Host genotypes grouped by their early season infection status and the locations of the four transplant populations included in this study. (*a*) *Plantago lanceolata* sentinel plants grouped by their infection status, (1) uninfected (2) single infection by *Plantago closterovirus, Plantago betapartitivirus* or *Plantago lanceolata latent virus* (*n* = 213), at the early season timepoint. The colours represent different host genotypes. (*b*) The locations of field experimental sites in the Åland Islands, Finland. ‘Uninfected*'* refers to uninfected individuals, ‘*Closterovirus*’ to *Plantago closterovirus*, ‘*Betapartitivirus*’ to *Plantago betapartitivirus* and ‘PlLV’ to *Plantago lanceolata latent virus*.

**Figure 2.**
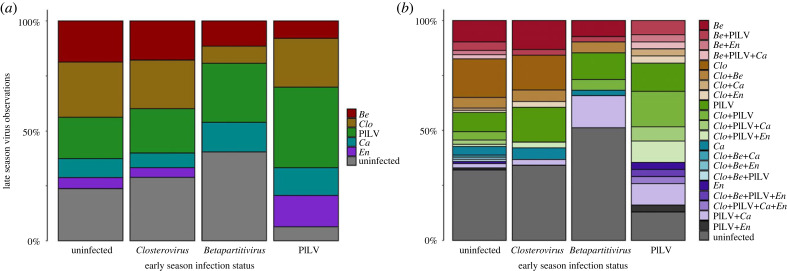
Late season viral occurrences grouped according to the early season infection status of host plants. (*a*) Prevalences of the five focal viruses during the late season timeperiod in transplanted sentinel *Plantago lanceolata* plants. Viral prevalences are grouped by the host's early season infection status. (*b*) Co-occurrences of the five focal viruses during the late season timeperiod, grouped by the host's early season infection status. ‘*En*’ refers to *Plantago enamovirus,* ‘*Ca*’ to *Plantago latent caulimovirus*, ‘*Clo*’ or ‘*Closterovirus*’ to *Plantago closterovirus,* PlLV to *Plantago lanceolata latent virus* and ‘*Be*’ or ‘*Betapartitivirus*’ to *Plantago betapartitivirus*.

In the early season timepoint, 39% of the plants (*n* = 320) were infected by a single virus, 14% harboured a co-infection and 47% were uninfected (more detailed description of all viral infections in the early season timepoint in electronic supplementary material, table S2). To investigate how priority effects shape the late season viral community, we selected a total of 110 plant individuals with a single virus infection by PlLV (*n* = 31), *Closterovirus* (*n* = 38) or *Betapartitivirus* (*n* = 41; figure 1) at the early season timepoint and, for comparison, 103 plant individuals with no viral infection at that time. Hence, our study included a total of 213 plant individuals, which were furthermore selected to represent the four plant genotypes as equally as possible (genotypes; 511_14: *n* = 45, 609_19: *n* = 53, 2818_6: *n* = 54, 4_13: *n* = 61). Among the pooled late season samples (*n* = 639 pooled viral occurrences across the three late season sampling dates), 33% of the sentinel plants were uninfected, 35% had a single infection and 31% were co-infected by multiple viruses (figure 2). We detected all five focal viruses in the late season timeperiod; PlLV was the most abundant virus, accounting for 35% of all infections in sentinel plants, whereas *Enamovirus* was least abundant, with an 8% infection frequency. The late season viral communities were variable in composition, ranging from single infections to co-infections with as many as four viruses. We detected 21 different virus combinations in total (figure 2*b*).

### (b) Analysis of viral priority effects

Late season viral occurrences were well predicted, although model performance varied among the five focal viruses. PlLV occurrences were most reliably predicted and *Betapartitivirus* occurrences were least reliably predicted (table 1). The mean explanatory power of our models in terms of Tjur R^2^ was 0.23 (range among viruses 0.12–0.56) and the mean AUC was 0.86 (range 0.80–0.92). Model predictive power based on 10-fold cross-validation was slightly lower, with a mean Tjur *R*^2^ of 0.16 (range among viruses 0.05–0.52) and a mean AUC of 0.71 (range 0.62–0.84) (table 1). Lastly, the explanatory power of our models from each of the late season sampling timepoints (T2–T4) in terms of Tjur *R*^2^ were lower than when compared with the pooled model and varied among the five focal viruses (see details in electronic supplementary material, table S3). Mean AUC values resembled those of the pooled model (T2 = 0.89, T3 = 0.92 and T4 = 0.89; electronic supplementary material, table S3).

**Table 1.** Explanatory performance and predictive performance, based on 10-fold cross validation, of late season occupancy models of five focal viruses in terms of Tjur *R*^2^ and AUC.

model	model explanatory performance		model predictive performance with 10-fold cross validation (cv)	
response variable*	Tjur *R*^2^	AUC	Tjur *R*^2^ (cv)	AUC (cv)
*Closterovirus*	0.19	0.84	0.07	0.62
*Enamovirus*	0.14	0.9	0.08	0.75
*Betapartitivirus*	0.12	0.8	0.05	0.64
PlLV	0.56	0.92	0.52	0.84
*Caulimovirus*	0.15	0.83	0.1	0.71

**Figure 3.**
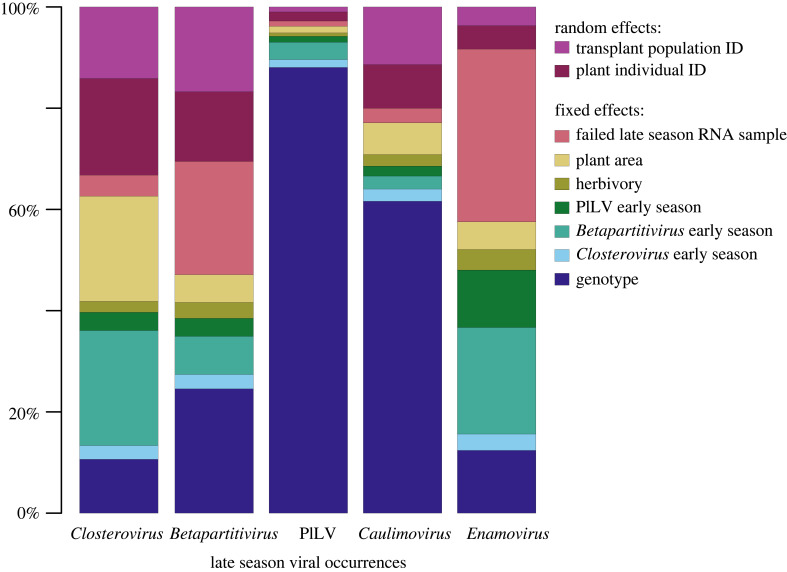
Proportion of variance explained (out of the total variation explained by the model) by the fixed and random effects in the HMSC model of the late season occurrences of the five focal viruses in the transplant experiment. The nine variables explaining the late season occurrences of each virus (in columns) were transplant population and plant individual ID (random effects), host plant genotype, host plant size, the presence/absence of herbivory, the early summer occurrences of three of the viruses (*Plantago closterovirus*, *Plantago betapartitivirus* and *Plantago lanceolata latent virus*) and differences in RNA viral sampling efficiency. Here, ‘*Enamovirus*’ refers to *Plantago enamovirus,* ‘*Caulimovirus*’ to *Plantago latent caulimovirus*, ‘*Closterovirus*’ to *Plantago closterovirus,* PlLV to *Plantago lanceolata latent virus* and ‘*Betapartitivirus*’ to *Plantago betapartitivirus*.

In terms of contributions to explained variation, host plant genotype was the strongest determinant of mean late season viral occurrences explaining 39% of variance (figure 3 and table 2). This was followed by the early season infection status of the host, which explained 18% of the variance in late season viral occurrences. Plant size and the presence/absence of herbivory also contributed to explained variance (8% and 2%, respectively, figure 3 and table 2). Finally, the number of failed RNA samples during the late season explained 13% of variance. The results for the models of each sampling timepoint individually (T2–T4) showed similar results, where the host genotype explained most of the variation, however, there were some differences between timepoints. Model results from sampling timepoint 4 were very similar to those of the pooled model (see electronic supplementary material, figure S1 showing explained variance of the total variance explained by all variables in the T2–T4 models).

**Table 2.** Posterior mean and 95% credible intervals for the model variance partitioning. Response variables in the model were the late season occurrences of the five focal viruses and the parameters were the fixed and random effect covariates included in the model. Fixed effects in the model were: host plant genotype, early season infection status of the host (*Plantago closterovirus*, *Plantago betapartitivirus* or *Plantago lanceolata latent virus* single infection), signs of herbivory, plant area and failed late season RNA sampling. Random effects were the plant individual ID and transplant population ID. In the table ‘*Enamovirus*’ refers to *Plantago enamovirus,* ‘*Caulimovirus*’ to *Plantago latent caulimovirus*, ‘*Closterovirus*’ to *Plantago closterovirus,* PlLV to *Plantago lanceolata latent virus* and ‘*Betapartitivirus*’ to *Plantago betapartitivirus.*

response (late season viral occurrence)	parameter	mean	95% credible interval	
			lower 2.5%	upper 97.5%
*Closterovirus*	genotype	10.5	0.5	34
*Closterovirus*	closterovirus early season	2.7	0	13.1
*Closterovirus*	betapartitivirus early season	23.2	1.7	58.2
*Closterovirus*	PlLV early season	3.5	0	18.1
*Closterovirus*	herbivory	2.1	0	10.8
*Closterovirus*	plant area	20.7	1.1	52.4
*Closterovirus*	failed late season RNA sample	4	0	20.1
*Closterovirus*	plant individual ID	19.1	0.1	82.5
*Closterovirus*	transplant population ID	14.2	0.1	51.6
*Enamovirus*	genotype	12.3	1.1	33.6
*Enamovirus*	closterovirus early season	3.2	0	15.7
*Enamovirus*	betapartitivirus early season	21	1.1	57.3
*Enamovirus*	PlLV early season	11.3	0.4	34.9
*Enamovirus*	herbivory	4	0	14.9
*Enamovirus*	plant area	5.4	0	24.3
*Enamovirus*	failed late season RNA sample	34.5	1.9	77.9
*Enamovirus*	plant individual ID	4.6	0	25.2
*Enamovirus*	transplant population ID	3.7	0	20.3
*Betapartitivirus*	genotype	24.7	4	56.4
*Betapartitivirus*	closterovirus early season	2.8	0	12.6
*Betapartitivirus*	betapartitivirus early season	7.5	0.1	27.6
*Betapartitivirus*	PlLV early season	3.5	0	17.4
*Betapartitivirus*	herbivory	2.9	0	12.5
*Betapartitivirus*	plant area	5.4	0	22.3
*Betapartitivirus*	failed late season RNA sample	22.7	1	57.9
*Betapartitivirus*	plant individual ID	13.7	0.1	74.6
*Betapartitivirus*	transplant population ID	16.8	0.2	56.2
PlLV	genotype	88	74.2	95.7
PlLV	closterovirus early season	1.6	0	6.3
PlLV	betapartitivirus early season	3.4	0	10
PlLV	PlLV early season	1.2	0	5.4
PlLV	herbivory	0.7	0	3.5
PlLV	plant area	1.3	0	7.2
PlLV	failed late season RNA sample	1	0	4.8
PlLV	plant individual ID	1.8	0	10.9
PlLV	transplant population ID	1	0	5.5
*Caulimovirus*	genotype	61.7	29.3	85.3
*Caulimovirus*	closterovirus early season	2.4	0	11.6
*Caulimovirus*	betapartitivirus early season	2.6	0	12.7
*Caulimovirus*	PlLV early season	1.9	0	10.5
*Caulimovirus*	herbivory	2.3	0	10.9
*Caulimovirus*	plant area	6.3	0	23.4
*Caulimovirus*	failed late season RNA sample	2.8	0	15.1
*Caulimovirus*	plant individual ID	8.6	0.1	45.9
*Caulimovirus*	transplant population ID	11.4	0.1	42.1

**Figure 4.**
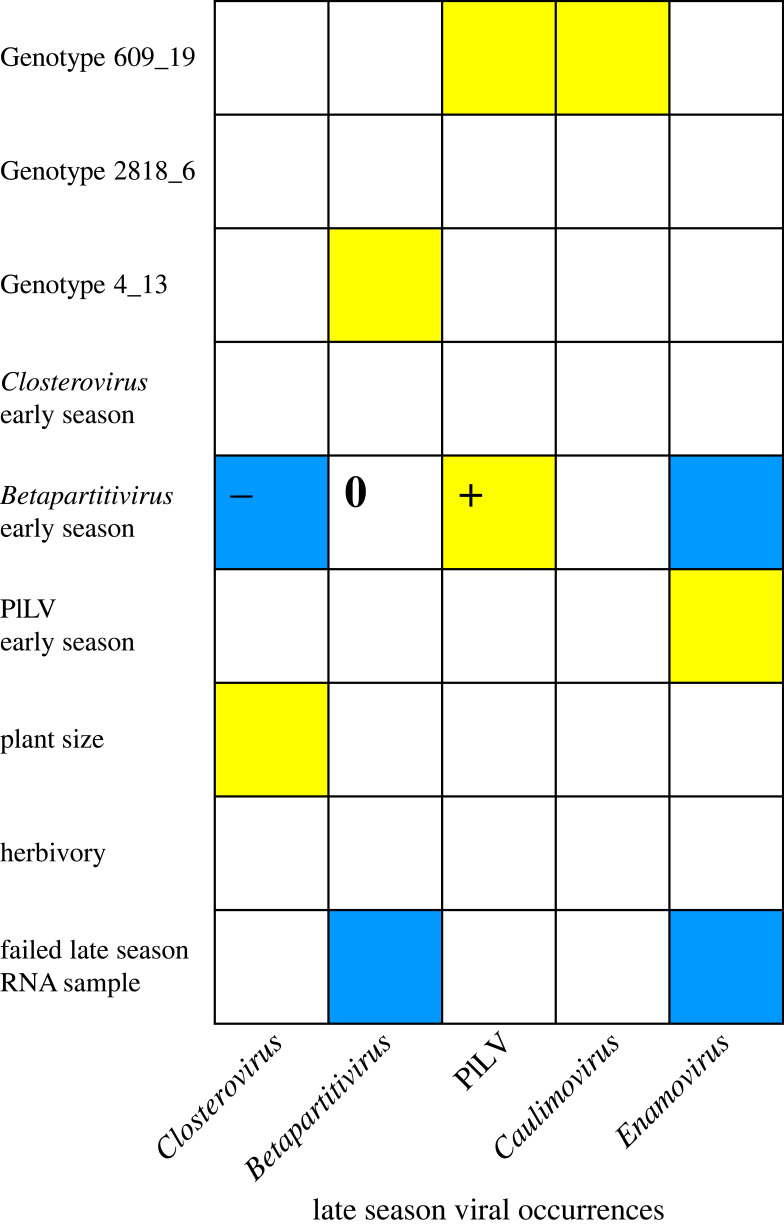
Predicted responses of the late season occurrences of five focal viruses to host genotype, early season infection status, herbivory and size, as well as the number of failed late season RNA samples, in a transplant experiment on *Plantago lanceolata*. The colours indicate predicted positive (yellow, ‘+’) and negative (blue, ‘−’) late season responses of each virus to the fixed effect covariates in the model that received >95% posterior support. Predicted responses to plant genotypes 609_19, 2818_6 and 4_13 are illustrated relative to baseline genotype 511_14. ‘*Enamovirus*’ refers to *Plantago enamovirus,* ‘*Caulimovirus*’ to *Plantago latent caulimovirus*, ‘*Closterovirus*’ to *Plantago closterovirus,* PlLV to *Plantago lanceolata latent virus* and ‘*Betapartitivirus*’ to *Plantago betapartitivirus*. The mean values of the beta parameter estimates and their credible intervals are in electronic supplementary material, table S4.

The effects of host genotype on late season viral occurrences varied strongly among viruses. Genotype effects were pronounced for the two DNA viruses, PlLV and *Caulimovirus* (88% and 62% of variance explained by genotype, respectively; figure 3 and table 2), both of which were favored by host genotype 609_19 (figure 4). Host genotype played a less pronounced role in defining the colonization patterns of the three RNA viruses, *Betapartitivirus*, *Closterovirus* and *Enamoviru*s (figure 3 and table 2). However, genotype 4_13 was more likely to host *Betapartitivirus* infections than the other genotypes (figure 4).

The five focal viruses also differed in their sensitivity to prior viral infection, as indicated by the summed proportion of variance explained by prior infection by any early season virus (in decreasing order: *Enamovirus* 36%*, Closterovirus* 29%, *Betaparitivirus* 14%, *Caulimovirus* 7% and PlLV 6%; figure 3 and table 2). Infection by *Betapartitivirus* at the beginning of the season had the greatest impact on subsequent colonization dynamics as revealed by strongly supported (*p* > 0.95) negative and positive effects on late season viral colonization. Plant individuals colonized early by *Betapartitivirus* were less likely to host *Closterovirus* or *Enamoviru*s in the late season, and were more likely to host PlLV (figure 4; see also estimated beta-parameter values and their credible intervals in electronic supplementary material, table S4 and figure S2). Furthermore, the presence of PlLV in the early season increased the probability of detecting *Enamoviru*s in the late season (figure 4; electronic supplementary material, table S4).

**Figure 5.**
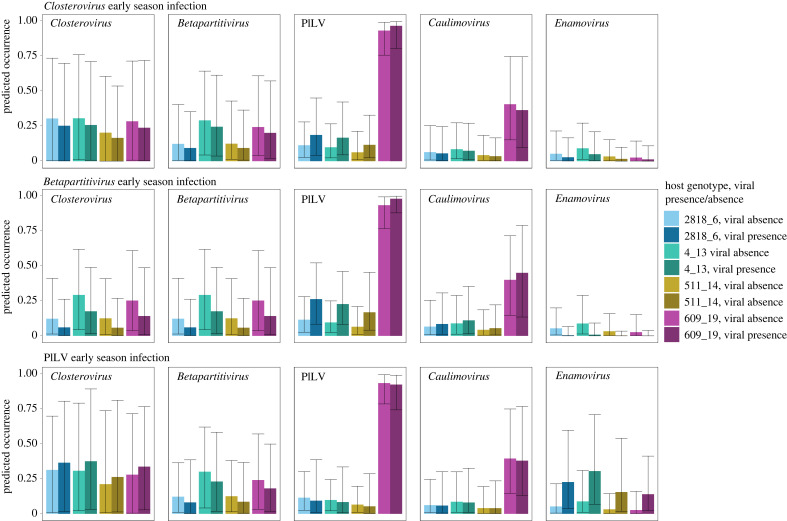
Marginal effect predictions of early season infection status on late season virus prevalence by host genotype. Predicted effect of early season infection status on late season prevalence of the five focal viruses on four genotypes of *Plantago lanceolata* in a transplant experiment. The predicted late season prevalences of the five focal viruses (response variables) per host plant genotype are illustrated as a function of early season viral infection status (in rows) by *Plantago betapartitivirus*, *Plantago closterovirus* or *Plantago lanceolata latent virus* (binary fixed effect predictors). Each distinct colour represents a plant host genotype, and within these, the lighter tone represents early season viral absence and the darker tone viral presence (colour codes: dark blue = genotype 2818_6/viral presence, light blue = genotype 2818_6/viral absence; dark green = 4_13/viral presence, light green = 4_13/viral absence; dark yellow = 511_14/viral presence, light yellow = 511_14/viral absence; dark purple = 609_19/viral presence, light purple = 609_19/viral absence). The whiskers represent the bounds of 95% credible intervals of the median prediction. Orange stars highlight cases in which there is strong posterior support for a directional effect of early season viral infection status on the late season prevalence of a focal virus. In the figure ‘*Enamovirus*’ refers to *Plantago enamovirus,* ‘*Caulimovirus*’ to *Plantago latent caulimovirus*, ‘*Closterovirus*’ to *Plantago closterovirus,* PlLV to *Plantago lanceolata latent virus* and ‘*Betapartitivirus*’ to *Plantago betapartitivirus*.

To illustrate the predicted effects of early season infection status on late season viral prevalence per host plant genotype, we generated marginal effects prediction plots based on the HMSC model, where all covariates apart from the focal early season virus and host genotype were set at their mean value in the dataset (figure 5; see electronic supplementary material, figure S3 for the predicted effects of the other covariates). As expected, based on the predicted responses to each viral covariate described above (figure 3) initial infection by *Betapartitivirus* is predicted to facilitate sequential PlLV infection. In addition, inhibitory priority effects of *Betapartitivirus* on both *Closterovirus* and *Enamovirus* are predicted*.* Early colonization by PlLV, in turn, is predicted to facilitate sequential infection by *Enamovirus.* Plant genotype furthermore affects the absolute predicted prevalence of each virus, for example, host genotype 609_19 is predicted to harbour PlLV at a very high prevalence.

Host plant size explained 8% of variance (figure 3 and table 2) and was predicted to have a positive effect on the late summer occurrence probability of *Closterovirus* (figure 4). Herbivory explained least variance in our model, 2%, and was not connected to any specific host genotype (figure 4). Lastly, the number of failed RNA samples during the late summer explained 13% of variance, and was predicted to have a negative effect—as anticipated—on the detection of late season occurrence probabilities of two of the three RNA viruses, *Enamoviru*s and *Betapartitivirus* (figure 4).

We also detected some residual structure in late season viral occurrences that was unexplained by the fixed effect covariates, both among plant individuals and among the four populations, mean variance explained 10% and 9%, respectively (figure 3 and table 2). This unexplained structure could reflect the effects of (i) missing ecological drivers, or (ii) structure arising from direct positive or negative interactions between the viruses that we did not capture with our three early season virus variables. However, we do not see any strongly supported positive or negative residual correlations between the late season occurrences of the viruses that would back the second interpretation.

## 4. Discussion

Prior research has demonstrated that priority effects—defined by the sequence of arrival of species—shape the trajectory of community assembly in many systems [51,83,84]. Much like free-living organisms, viruses, and other pathogens, can form diverse and complex assemblages but this assembly process takes place within their hosts. Our results suggest that priority effects can arise in viral assemblages as a result of sequential host infection. By combining a transplant experiment with a hierarchical joint model of viral occurrences, we were able to detect both facilitative and inhibitory priority effects among viruses, depending on which virus first infected the host early in the season. Furthermore, by transplanting naïve host clones, we were able to control for host genotypic variation and most importantly the timing of initial viral infection. However, we acknowledge that our study does not address the effects of potential later season co-infections on viral colonization dynamics. Our findings nonetheless highlight the importance of biotic interactions—encompassing both the arrival order of viruses and host genotype—in determining viral community assembly.

Our study focused on five *Pl. lanceolata* infecting viruses that previous studies have shown to be among the most abundant viruses in this study system [67]. The transplanted sentinel plants harboured relatively high infection rates; 53% of the plants individuals were infected by one or more viruses early in the season and 66% in the late season time period. The high virus prevalence in this wild plant system resembles that of agricultural settings, where virus-free plants acquire virus infections during the growing season, highlighting the importance of seasonal transmission for viruses [85,86]. Our model results indicate that, after accounting for the effect of host genotype, the most important factor explaining viral colonization patterns in the late season was the early season viral infection status of the host. We found that early infection with *Betapartitivirus* inhibited subsequent colonization by *Closterovirus* and *Enamoviru*s but facilitated PlLV infection. Moreover, PlLV colonization early in the season facilitated *Enamoviru*s infection later in the season. Interestingly, we did not find clear indications that individual viruses are consistently facilitative or inhibitory. *Betapartitivirus* was involved both in inhibitory and facilitative priority effects. *Betapartitivirus* belongs to the family Partitiviridae, which is known to cause persistent viral infections [87] and to co-occur with other dsRNA viruses [88], suggesting that the role of *Betapartitivirus* in community assembly is often likely to be facilitative. Viruses from the Partitiviridae family are generally believed to be transmitted vertically through the germline [87]. While our cloned sentinel plants were initially virus-free, we detected *Betapartitivirus* infections in the late season, suggesting a non-vertical transmission route. In addition to plants, *Betapartitivirus* can also infect fungi and protozoa, and hence, a possible transmission route could be from fungi to plants [77,89]. PlLV is more comprehensively characterized than the other four viruses studied here and, based on our previous research, is one of the most prevalent viruses infecting *Pl. lanceolata* in the Åland Islands. Given the overall prevalence of PlLV in this system [24,65,66], and its tendency for facilitation, PILV may be an important biotic promotor of within-host viral diversity. By focusing on the five focal *Pl. lanceolata* infecting viruses for which we have developed PCR primers, we acknowledge that some viruses were likely excluded from the scope of this study. The effects of other unidentified viruses, as well as other micro-organisms, could of course contribute to some of the unexplained variation in our model.

In addition to viral priority effects, we detected differences among *Pl. lanceolata* genotypes in their probability of late season viral infection which are in line with earlier findings in this system [10]. Plant genotype was clearly the strongest determinant of late season viral colonization patterns, explaining on average 39% of the variation in our HMSC model (figure 3). We found similar genotype effect in our models for each sampling timepoint individually (electronic supplementary material, figure S1). Furthermore, the magnitude and direction of viral facilitative or inhibitory priority effects differed among genotypes (figures 4 and 5). The effect of genotype was most pronounced for genotypes 609_19 and 4_13, which were susceptible to PlLV, *Caulimovirus* and *Betapartitivirus*. Similar host genotypic effects on infection probabilities by viruses and other parasites have been detected across a range of host species [10,90–92]. By using cloned individuals, we were able to account for genotypic variation and to ensure homogenous host material. Previous studies from the Åland Islands system have shown *Pl. lanceolata* genotypes to harbour extensive diversity in resistance to powdery mildew *Po. plantaginis* [29,93,94]. This observation, together with our new results, suggests that genotypic variation in resistance in *Pl. lanceolata* also holds true for viruses. As the sentinel plants were placed within natural *Pl. lanceolata* populations, viral prevalence in the transplant populations may also have influenced the observed viral occurrences [95,96]. There is a growing body of evidence that insect vectors may prefer hosts that are already infected, enhancing viral spread and promoting co-infections [35–38,97,98]. Also here, the effect of plant genotype may partially arise from the plant virus vectors preferring specific host genotypes.

Viruses have been traditionally thought of only as infective agents causing disease, and indeed viruses cause severe diseases in humans as well as in domestic animals and crops [99,100]. Viral disease outbreaks can result in humanitarian crises and significant economic losses [101,102]. However, recent advances in *virome* research suggest that, in fact, the role of viruses in ecosystems is multifaceted [56,103]. For example, viruses contribute to important ecosystem services such as nutrient cycling [104]. Furthermore, viral communities harbour a tremendous pool of genetic variety and, through infection, maintain variation in host organisms as well [105]. It is well established that co-infecting viruses may strongly impact viral infection progression within hosts, as well as transmission among hosts [32,106]. Hence, understanding the drivers of viral community assembly can help us to understand virus ecology and, more specifically, the role of viral communities in health and in disease [54,55]. To our knowledge, our study is the first field experiment to study viral priority effects in the wild. Our results highlight the importance of the infection history and genotype of the host individual in shaping viral community assembly, but also the importance of studying virus ecology at the community level. Our study shows that past infections may, in some cases, determine the course of future infections, bringing us one step closer to understanding the dynamics of viral communities.

## Data Availability

The data and R scripts used in this study have been submitted to GitHub (https://github.com/mirkkajones/ViralCommHmsc) and to Dryad (https://doi.org/10.5061/dryad.rr4xgxdf2) [107]. The data are provided in electronic supplementary material [108].
